# Supplementary material for: Are carbon emissions trading and green financial instruments synergistic? -Comprehensive quantitative research based on content analysis
Source: PLoS One. 2024 Mar 7;19(3):e0298601. doi: 10.1371/journal.pone.0298601 (PMC10919601; doi:10.1371/journal.pone.0298601)
Supplement: S1 Data — (ZIP) [file pone.0298601.s001.zip › Data/地级市Z_F工作报告.zip!/║╙▒▒/║Γ╦«╩╨/└┤╫╘╣1⁄2╓┌║┼í╛┬φ┐╦╩2╛▌═°í┐_2003.docx]

各位代表：

现在，我代表枣强县人民政府向大会作政府工作报告，请予审议，并请县政协各位委员和其他列席人员提出意见。

过去五年工作的简要回顾

过去的五年，是世纪之交的五年，也是枣强经济经受考验、实现突破性发展的重要时期。全县人民在县委的领导下，认真贯彻“三个代表”重要思想，奋发图强，扎实工作，努力克服各种困难和不利因素，开创了新世纪枣强经济和社会发展的新局面。

国民经济稳步增长，发展基础得到夯实。五年来，国内生产总值在高位运行的前提下，年均递增6%；财政收入在累计消化虚收6356万元的基础上，2002年完成12332万元，比1997年实际增长近一倍，质量提高，实力增强，为全县经济的健康发展奠定了基础。

农村经济全面发展，特色产业迅速壮大。立足枣强实际，确立了红枣、奶牛两大主导产业。通过强化引导，精心组织，使两大产业快速起步、迅速发展。到2002年，红枣业发展到7万亩、160万株；奶牛业从零起步，发展到3000头，建成养殖小区5个。种植结构进一步优化。粮经比例由75∶25调整到56∶44，农业产业化经营率达到30%，比1997年提高10个百分点。农业生产条件明显改善，全县旱涝保收田达到54万亩。顺利完成了农村税费改革和土地延包，确保了农村各项方针政策的落实。

县乡工业焕发生机，整体素质显著增强。把玻璃钢、皮毛作为主导产业和立县产业，集中力量，重点推进，两大产业的利税增幅连续两年保持在30%以上。2002年，玻璃钢产业新增摊点138家，上交税金1100多万元；皮毛产业新增摊点1000多家，上交税金2200多万元。园区建设步伐加快。2002年玻璃钢工业园区完成投资7300万元，新增入园企业8家，累计达到25家。皮毛工业园区迅速膨胀，两年来，累计投入资金上亿元，完成了“两纵四横”的路街建设，实现了“四通一平”，建成企业19家。两个园区框架基本搭起，功能日臻完善，已经成为全县经济发展的新平台。项目建设质量提高。恒润、瑞隆、科力、大有、富尔派、恒兴、竞佳等一批重点项目，规模大、起点高，技术、工艺达到了国内先进水平。县乡企业改制稳步推进。改制企业达到120多家，共盘活闲置资产2.6亿元，吸纳民间资本近亿元，为经济发展注入了新的活力。

发展环境不断改善，民营经济蒸蒸日上。以整治大营皮毛发展环境为突破口，刹风治乱，促进了全县发展环境的根本好转。同时，不断完善鼓励民营经济发展的各项政策措施，积极为民营企业排忧解难，调动了民营企业大干快上的积极性。2002年，全县民营经济实现销售收入32.9亿元，上交税金4600万元，分别是1997年的2.2倍和2.3倍。全县纳税百万元以上的民营企业达到9家，10万元以上的达到95家。民营经济的蓬勃发展，有力地促进了全县经济的快速增长，促进了职工就业和第三产业的繁荣。

基础设施日臻完善，县城面貌明显改观。坚持“经营城市”的理念，多方筹措资金，加快县城建设，新建和翻修改造了西环路、东环路、新华街、裕华路、胜利路等主干路街，改造了县城北出口，建设了文化休闲广场，综合治理了环境卫生，全面加强了市政建设，初步实现了“道路变宽，交通变畅，秩序变好，夜晚变亮”的目标。大营镇被评为“全国小城镇综合改革试点镇”。加会被省政府批准为建制镇。翻修了枣郑路、枣冀路，新建桥涵17座，交通条件进一步改善。高标准完成了农村电网改造工程，年购电量达到2.4亿度。全县固定电话装机总量达到6万门，移动电话用户达到3.9万户，分别是1997年的4.7倍和48倍。

双向开放取得新成绩，外贸出口快速增长。2002年，全县自营出口企业达到34家，比1997年增加29家。完成外贸出口668万美元，比1997年增长近10倍。成功举办了中国大营国际皮草交易会、中国玻璃钢发展枣强现场会和全国玻璃钢可持续发展现场会，大大提高了枣强的知名度。

财税金融平稳运行。扎实推进财税体制改革，强化征管，堵塞漏洞，确保应收尽收；认真落实财政集中支付、政府采购和收支两条线规定，严格控制支出，实现了收支基本平衡，确保了公教人员工资发放。全县存款余额达到31亿元，贷款余额17亿元，实现了存贷双增。

科教文卫等社会事业全面发展。五年来，共开发省级以上新产品69项，玻璃钢工业园区被确定为全省“十大民营科技园区”之一，省玻璃钢研究所、化工研究所、空调研究所、玻璃钢生产力促进中心相继落户枣强。全面推行教育内部改革，顺利通过了省普九复检。建成了县医院新病房楼，就医条件进一步改善。环保和计划生育圆满完成任务目标。人民武装、社会保障、广播电视、民政、国土、体育、统计、审计、物价、档案等社会各项事业取得新进展。

民主法制和精神文明建设进一步加强。以“三个代表”学教活动为契机，群众性精神文明创建活动扎实开展。大力推行“四公开”，基层民主政治建设迈出新步伐。人大代表建议和政协委员提案办理质量不断提高，按时办结率达到100%。不断强化信访工作，认真开展矛盾纠纷排查，有效地化解了各种不安定因素。扎实开展严打整治斗争，有力地打击了各种违法犯罪活动。全县政治安定、社会稳定的大好局面进一步巩固。

各位代表，经过全县人民的共同努力，枣强面貌正在发生着重大变化，呈现出人心凝聚、经济提速、亮点增多、事业发展的喜人局面。这些成绩的取得，是县委正确领导、人大、政协积极监督、鼎力支持的结果，是全县广大干部群众团结奋斗的结果。在此，我谨代表县政府向各位代表、各位委员，并通过你们向全县广大干部群众和所有关心、支持、帮助我县发展的朋友们、同志们，表示崇高的敬意和衷心的感谢！

总结过去的工作，我们的主要体会是：

──必须坚持解放思想、实事求是、与时俱进的思想路线。在解放思想中统一思想，在与时俱进中创新发展，积极探索具有时代特征、体现枣强特色的发展路子。

──必须把加快发展作为“第一要务”，紧紧抓住经济建设不放松。靠发展统一思想，靠发展凝聚人心，靠发展解决前进道路上的困难和问题。

──必须坚持从实际出发，创造性地开展工作。立足枣强实际，充分挖掘发展潜力，充分发挥自身优势，充分调动各种积极因素，不断开创各项工作新局面。

──必须坚持求真务实、狠抓落实的工作作风。不搞形式主义，不做表面文章，靠实实在在的工作，干实实在在的事业，求实实在在的效益。

──必须坚持重点突破，带动全局。紧紧扭住影响枣强发展的关键环节和重点工作，全力攻坚，以重点工作的突破，活跃和带动工作全局。

──必须坚持执政为民，真心实意地为群众办实事。千方百计解决群众关心的热点、难点问题，一切为了群众，一切依靠群众，加快枣强各项事业发展。

在肯定成绩的同时，我们也清醒地看到，全县经济和社会发展中还存在着一些不容忽视的矛盾和问题：经济发展速度还不够快，质量还不够高，综合经济实力有待于进一步增强；农业主导产业规模小，农民增收缓慢；立县、立乡的大项目少，骨干企业少，工业强县的任务还十分艰巨；对外开放水平不高；城镇基础设施建设相对滞后；日益增长的发展需要与财政增长的矛盾还比较突出；干部队伍的思想观念、工作作风、办事效率与人民群众的要求还有一定差距。对此，必须高度重视，采取有效措施，努力加以解决。

对今后五年指导思想和奋斗目标的建议

党的十六大提出了到2020年全面建设小康社会的宏伟目标，同时明确指出：新世纪头二十年，是一个必须紧紧抓住并且可以大有作为的重要战略机遇期。抓住机遇，乘势而上，建设一个惠及全县人民的更高水平的小康社会，是全县人民的共同期盼，也是时代赋于政府的神圣使命。

今后五年，是加快枣强发展，实现位次前移，为建设小康社会奠定基础的重要阶段，也是增创枣强新优势，增强枣强竞争力，在区域经济竞争格局中赢得有利位置的关键时期。我们建议，新一届政府要按照十六大提出的“发展要有新思路，改革要有新突破，开放要有新局面，各项工作要有新举措”的总体要求，高扬“树正气、讲团结、求发展”的主旋律，团结和带领全县人民聚精会神搞建设，一心一意谋发展，向着更新更高的目标迈进。总的指导思想是：以十六大精神和“三个代表”重要思想为指导，以全面建设小康社会总揽全局，以加快发展、富民强县为第一要务，大力发展四龙经济、民营经济和开放型经济，突出项目建设、园区建设、县城建设、环境建设四个重点，加快工业化、农业产业化和城镇化进程，改善人民生活，确保社会稳定，实现“五个明显变化”，推进国民经济跨跃式发展和社会全面进步。

五个明显变化：一是经济实力明显增强。到2007年，全县国内生产总值力争在2000年的基础上翻一番。二是特色产业明显壮大。玻璃钢、皮毛两大产业在不断扩大规模的基础上，运用高新技术，加大改造力度，调整产品结构，建成国内一流的产业基地；红枣、奶牛两大产业在加快规模扩张的同时，逐步实现生产、加工、销售一条龙，使之成为农民增收的重要来源。三是农业结构明显优化。全县农业产业化经营率达到50%以上，蔬菜、果品、畜产品无公害认定率分别达到100%、90%和80%。四是城镇面貌明显改观。县城面积达到22平方公里。五是人民生活明显改善。城乡居民收入大幅增长，社会保障体系进一步完善。

实现上述目标，关键是加快经济发展，重点是实施“四大战略”：

一是工业强县战略。全面建设小康社会，工业是关键。必须做到精力向工业集中，政策向工业倾斜，重点向工业转移。积极采用新技术、新工艺改造传统产业，深化企业改革，加快建立现代企业制度，努力实现科技创新、机制创新和管理创新。突出抓好玻璃钢、皮毛两个工业园区，把两大园区建成产业发展的龙头、对外开放的窗口、工业腾飞的基地。到2007年，全县利税超千万的企业达到10家以上。

二是民营富县战略。民营经济是最具活力的经济，其发展快慢决定着全面建设小康社会的进程。要千方百计为民营经济提供理想的发展平台，创造广阔的发展空间，集民力、民资、民智，加快枣强发展。到2007年，全县民营经济占国民经济的比重要达到90%以上。

三是项目立县战略。项目是调整结构、加快发展的载体，是推进工业化、农业产业化和城镇化的关键，必须把项目建设作为一项长期的战略任务，坚持不懈地抓紧抓好。要围绕国家产业政策，谋划实施一批规模大、附加值高、产业链条长的农业产业化项目；谋划实施一批市场前景好、科技含量高、带动作用强的工业技改项目；谋划实施一批完善县城功能、促进经济发展的基础设施项目，加大跑办力度，力争挤进国家和省市计划“笼子”。

四是开放兴县战略。适应加入WTO和经济全球化的新形势，向开放要出路，靠开放促发展，按照“利用人缘优势、发挥产业优势、构筑环境优势、营造招商优势”的思路，全力推进双向开放。

对2003年政府工作的建议

2003年是全面贯彻党的十六大精神的第一年，也是争先晋位、跻入全市先进行列的决战年。做好今年的工作，意义特别重大。全县国民经济和社会发展的主要预期目标是：国内生产总值增长10%，全社会固定资产投资增长17%以上，财政收入力争增长10%。重点做好八个方面的工作：

一、以民营经济为主体，以园区建设为重点，大力实施“工业强县”战略，加快推进工业化进程

抓好“两园一带、百村万户”，全力推进玻璃钢、皮毛两大龙型经济上规模、上水平。“两园”，就是玻璃钢工业园区和皮毛工业园区。玻璃钢工业园区重点向北向西推进，完成两条路街建设，形成“两纵四横”格局，新建企业8家，完成总投资6000万元。皮毛工业园区继续抓好道路延伸及配套设施建设，新建企业21家，完成总投资5000万元。“一带”，就是大营、新屯皮毛产业带，逐步消灭皮毛空白村，拓展产业区域，壮大产业规模，推进“营皮北扩”。“百村万户”，就是以玻璃钢、皮毛两大产业为依托，加快专业村、专业户建设。经过一两年的努力，使纳税10万元以上的专业村达到100个，年人均收入万元以上的户新增1万个，在全县形成点线面结合、辐射范围广的产业经济圈。

大力发展民营经济。一是浓厚氛围。大力宣传民营经济的地位和作用，在全社会营造“创业有功，纳税光荣”的浓厚氛围。二是加强引导。引导民营企业空白村，选准产业，加快启动；引导有一定实力的企业，向两个园区集中；引导骨干企业进一步做大做强，向规模企业发展。三是搞好服务。建立政府部门与民营企业联系、协调、服务制度，切实帮助民营企业解决生产经营中的困难和问题，促进民营企业健康发展。全年个体私营经济新增摊点1500家，产值、利税比上年增长20%以上。

搞好县乡企业改革、改制。继续按照“一退两转三买断”的原则，严格程序，规范运作，一厂一策，稳妥推进，确保改革一个，成功一个。帮助改制企业进一步强化管理，增加投入，加快发展，提高市场竞争力。

二、以农民增收为核心，以高效农业为重点，加快结构调整，推进我县由农业大县向农业强县转变

继续大力发展红枣、奶牛两大产业，加快农业产业化进程。红枣业以密植、片林为主，以马莲小枣为主，适当调整品种，确保全年育苗不少于1000亩，种植枣树达到160万株。加强对现有枣树的管护，提高成活率。同时，继续以确权绿化为突破口，发展速生丰产林1000万株，完成育苗5000亩。奶牛业以养殖小区为重点，空白乡镇抓启动，现有小区上规模。全县新增奶牛存栏3000头，总数达到6000头， 新建300头以上的标准养殖小区5个，抓好胚胎移植试点，建成奶业发展服务中心。围绕两大产业发展，突出做好三项工作：一是通过多种形式，大力宣传我县发展红枣、奶牛的优势和广阔前景，宣传县委、县政府的优惠政策，使发展两大产业成为广大农民的自觉行动。二是继续对两大产业实行政策倾斜和重点扶持，充分调动县内外客商和广大农民群众发展两大产业的积极性。三是充分发挥林业、畜牧等部门的职能作用，有针对性地搞好技术指导和销售服务，切实帮助群众解决各种实际问题。

加快“三个体系”建设，提高农业市场化程度。一是农业社会化服务体系。大力发展各种类型的农民合作组织，引导农民调结构、拓市场、促增收。二是农业科技推广体系。抓好优良品种的引进、繁育、推广，加快实用技术的开发和应用。三是农业标准化生产体系。按照国家“无公害食品行动”计划，组织好果品、蔬菜和畜禽产品的标准化生产，积极争取绿色标识认证。

加强农田水利建设，改善农业生产条件。坚持开源与节流并重，全年新打深浅机井500眼，铺设防渗管道10万米。继续抓好引黄配套工程，增强引蓄水能力。进一步加大农业综合开发力度，争取更多的项目，提高开发水平。

认真落实党在农村的基本政策。抓好土地承包法的宣传贯彻，稳定农村土地承包。巩固农村税费改革成果，推进配套改革，确保农村经济发展和社会稳定。

三、深入开展“招商引资年”活动，大力发展开放型经济

浓厚开放氛围。县委七届八次全会把今年确定为“招商引资年”，必须下大力做好开放文章，把开放贯穿到各项工作中去，在全县营造“上下左右齐动员，千军万马搞开放”的局面，形成“抓开放、促发展、求突破”的合力。

加强环境建设。在完善硬环境的同时，重点优化软环境，大力推行“零干扰招商、一站式服务”，简化审批手续，提高办事效率，充分发挥经济“110”作用，防止“三乱”反弹，积极营造公平规范的法制环境、优惠透明的政策环境、高效优质的服务环境。

改进招商方式。树立“县外即外”的招商观念，坚持内资、外资并举，“走出去”与“引进来”结合，以企业为主体，积极开展小团队招商、专业招商、合作招商和网上招商，在引进资金、项目、技术、人才的同时，注重引进先进的经营理念。精心组织企业参加意大利米兰国际皮草展览会和丹麦毛皮拍卖会，办好2003年中国大营国际皮草交易会，力争取得更加丰硕的成果。全年引进外资确保完成550万美元。

扩大外贸出口。在巩固扩大皮毛、化工等优势产品出口的同时，大力开发新的出口产品。力争全年新增自营出口企业15家，实现外贸出口800万美元。

四、全力推进项目建设，增强经济发展后劲

加快发展最终要落实在项目上，必须始终抓住项目建设不放松。工业，重点抓好两个园区的29个项目，确保年内建成投产。农业，重点抓好土地整理、退耕还林匹配、奶牛基地等项目，确保完成年度投资计划。基础设施建设，重点抓好县城、大营两个污水处理厂以及交通、电力、通讯等重点项目建设，加大跑办力度，争取早立项、早批复、早建设、早见效。对已经投产的项目，要切实抓好管理，争取最佳效益。全县谋划、建设投资100万元以上的项目力争达到103个，其中500万元以上的17个。

五、加快县城建设，提高城镇化水平

抓好绿化美化。以主干街道和县城出入口为重点，坚持一路一树、一路一灯、一路一景，突出特色，加大绿化美化力度。搞好胜利路、裕华路、富强路、人民街等道路的绿化改造，抓好卫千渠两侧的绿地建设和单位庭院绿化，让县城“绿起来、亮起来、美起来”。

搞好城市经营。按照国家有关规定，全部开放城镇建设投资领域，广泛吸引外资、内资、民资，参与城镇建设，推进城镇建设市场化、产业化、社会化。

强化县城管理。全面落实《城市管理实施办法》，坚决杜绝乱占、乱建，严格门前“四自五包”责任制，综合整治“脏、乱、差”。加强宣传教育，提高全民城市意识。

加快建设步伐。以县城和大营镇为重点，进一步放宽政策，降低门槛，吸引大户进城、农民进城，壮大城镇经济，膨胀城镇规模，带动三产发展，促进市场繁荣。

六、努力做好财税金融工作，实现财政状况的明显好转

组织好财政税收。坚持依法治税，大力推行税务公开，健全协税护税组织，严厉打击偷、逃、骗税行为，确保应收尽收。认真落实财政综合预算、集中支付、政府采购和收支两条线制度，强化预算外资金管理。严格支出顺序，确保公教人员工资发放，确保机关正常运转。做好金融工作。优化信贷投入，密切银企关系，促进金融和地方经济的协调发展。

七、加强社会主义政治文明和精神文明建设，推进社会全面进步

大力发展科教文卫事业。坚持科技兴工、科技兴农，加快培育科技型企业。进一步深化教育体制改革，大力推进素质教育，积极发展高中教育、职业技术教育和学前教育，提高教育教学质量。继续抓好科技、卫生、文化“三下乡”活动，提高群众科技文化素质。抓好新型农村合作医疗工作，解决农民因病致贫、返贫问题，促进卫生事业发展。

做好社会保障工作。扩大养老保险和低保覆盖面，进一步规范机关、事业单位养老保险，突出抓好社会保险费收缴和管理，确保养老金按时发放，确保八方面人员基本生活待遇的落实。

推进民主法制建设。自觉接受人大的法律监督、工作监督和政协的民主监督，推进决策民主化、科学化。加强基层民主政治建设，搞好“四五”普法，提高全民法制观念。整顿和规范市场经济秩序，净化市场环境。

全力维护社会稳定。深入开展严打斗争，严厉打击各种犯罪活动。坚决打击“法轮功”等邪教组织，做好教育转化工作。切实搞好社会治安综合治理，抓好信访和矛盾纠纷排查，及时消除不稳定因素。认真抓好安全生产，防止各类事故发生，确保人民生命财产安全。

此外，进一步做好计划生育、人民武装、广播电视、民政、人事、档案、审计、统计、体育等各项工作，促进经济与社会的协调发展。

八、加强和改进政府工作，更好地担负起发展重任

面对新形势、新任务，必须按照与时俱进的要求，切实加强政府自身建设。

转变职能，提高效率。适应新形势的要求，把政府职能转到经济调节、市场监管、社会管理和公共服务上来。加强公务员学习培训，提高公务员素质。强化改革创新意识，以更加开放的思路、更加开明的措施、更加灵活的办法，推动各项工作更加卓有成效地开展。

改进作风，狠抓落实。坚持深入基层，深入群众，关心群众疾苦，维护群众利益。加强政务督查，确保政令畅通。对重点工作、重要任务，实行目标管理，靠工作机制和工作制度，确保各项工作落到实处。

廉洁从政，树立形象。认真落实廉政建设责任制，做到组织领导到位，责任落实到位，政府班子成员和部门一把手要带头遵守廉洁自律的各项规定，以身作则，率先垂范。严肃财经纪律，纠正部门和行业不正之风，努力从源头上预防和治理腐败，树立良好的政府形象。

勤政为民，多办实事。做到权为民所用，情为民所系，利为民所谋。今年要力争办好8件实事：⑴建立新型农村合作医疗体系；⑵完成武馆线枣强北段大修；⑶开发建设枣强商城；⑷翻新改造市场街和建设南路中段；⑸完成胜利路便道和北环路的硬化工程；⑹新建5个通讯基站；⑺完成县城北出口、西出口的包装改造；⑻完成县城电网改造工程。

各位代表，回顾过去，我们豪情满怀；展望未来，我们信心百倍。让我们在市委、市政府和县委的领导下，高举邓小平理论伟大旗帜，全面贯彻“三个代表”重要思想，认真落实党的十六大精神，同心同德，团结拼搏，与时俱进，开拓创新，为胜利实现全面建设小康社会的宏伟目标而努力奋斗！
